# Is there a role for virtual reality in objective structured clinical examinations (OSCEs)?

**DOI:** 10.15694/mep.2019.000180.1

**Published:** 2019-09-17

**Authors:** Arjuna Thakker, Pooja Devani

**Affiliations:** 1 University of Birmingham College of Medical and Dental Sciences; 2 Leicester Royal Infirmary

**Keywords:** Virtual reality, medical education, medical student, OSCEs

## Abstract

This article was migrated. The article was marked as recommended.

Virtual reality is an area with fascinating possibilities. It has already revolutionised the entertainment industry, and over the last ten years, it has advanced similarly within the medical field. We have already seen virtual reality make a difference in a variety of ways. This ranges from improving the way surgical training is delivered, having a role in the management of chronic pain and now, it is even being considered in the treatment of psychiatric diseases. Currently, the application of virtual reality towards the training of health care professionals and medical education are concepts that are being explored. Within medical education and more specifically medical student training, the benefits of virtual reality have so far been limited to studies assessing its role in teaching. Very few studies exist looking at the role of virtual reality in the assessment of medical students. Therefore, in this article, we discuss the idea of utilising virtual reality for objective structured clinical examinations (OSCEs) and analyse the potential benefits and pitfalls of implementing this technology.

## Introduction

The idea of an altered reality only existing within the realms of science fiction has been long dispelled. Rather, reality distorting technology has been around in medicine for the past decade, having expanded from the entertainment, education and marketing industries. Currently, technology-based simulation can be split into two categories, augmented and virtual reality. Augmented reality integrates three-dimensional virtual objects into the user’s physical environment in real-time ( Berryman, 2012). Virtual reality simulates the user’s physical existence by providing multisensory, three-dimensional environments that enable them to become fully immersed in a simulated world ( Cipresso *et al*., 2018). The initial success of both augmented and virtual reality in medicine, for now, has been limited to their uses within the surgical field ( Li *et al*., 2017). However, due to constant improvements in technology, recent expansions into other domains of medicine have now been made possible. One field in which augmented and virtual reality is emerging to make an impact is medical education, in particular, medical student training ( Samadbeik *et al*., 2018).

With a particular focus on virtual reality rather than augmented, one of the most exciting visions for the future is the concept of developing simulated clinical stations for students as a tool for their examinations. Currently, objective structured clinical examinations (OSCEs) are the mainstay of clinical examinations for healthcare students. The idea of virtual reality simulations specifically designed for OSCEs is not a new concept, however. A study published in 2008 first demonstrated the efficacy of using computerised virtual patients as a tool for testing clinical reasoning and problem-solving amongst students. The results were favourable, with students showing a positive response to the new examination style ( Courteille *et al*., 2008). However, apart from this study, there have been no more studies to date assessing the use of virtual reality for OSCEs. So, in light of this, we ask the question, can a virtual reality designed OSCE station work?

## Discussion

The scope for integrating virtual reality with a specific OSCE station is dependent on what that particular station is intending to test. For example, in the UK, stations are specifically designed to test a range of skills, from acute care management, clinical skill procedures, and communication stations. For virtual reality to be a success, it would have to seamlessly integrate into these domains.

Arguably, the easiest domain to which virtual reality could be applied would be within acute care and management stations. These are stations designed to recreate simulated scenarios that test a candidate’s clinical acumen and responsiveness in an acute setting. This field has already been through one revolution, with the development of mannequin-based simulation, however, virtual reality now offers a more exciting prospect. At the forefront of pioneering the ultimate virtual reality experience in the UK, is a company called Oxford Medical Simulation. This company creates virtual scenarios specifically designed to immerse learners with interactive patients presenting in acutely unwell settings. Users must then manage the patient as in real-life, performing investigations, deciding the treatment and interacting with their interdisciplinary team. With this technology in place, it seems feasible to design a range of virtual scenarios that could simulate the curriculum of acute care stations for OSCE purposes across the UK. Moreover, these virtual scenarios used in the stations can be manipulated from candidate to candidate whilst still maintaining their accuracy and reproducibility. This makes it an attractive proposition to the rigid style of testing seen with OSCE stations. With such novel technology, there are of course hurdles to consider and overcome, mainly involving the logistics of such a system, such as training, dependability and cost. However, if virtual reality was to be incorporated into an examination, there would undoubtedly need to be preliminary tests and trials conducted to ensure the technology is both reliable and reproducible. Also, subsequent cost implications as well as potential savings (such as there no longer being a need for actors or expensive simulation mannequins) could be something that would be considered within a cost-effectiveness analysis.

The use of virtual reality to simulate the practical element of clinical skill procedures has already been explored in the field of dental education ( Huang *et al*., 2018, Joda *et al*., 2019). As of 2019, the potential benefits of two computerised virtual reality dental simulators are currently being explored across a handful of institutions around the world ( Roy *et al*., 2017). These simulators allow dental students to practise and refine their skills on computer-generated phantom heads rather than using the actual mechanical model. Virtual reality is then integrated with haptic technology (touch sensation) to produce reality-like scenarios ( Plessas, 2017). At the end of each simulation, an evaluation report and list of procedural errors can be produced for each student as a feedback tool ( Huang *et al*., 2018, Suebnukarn *et al*., 2011). With the technology already in place for dental education, the jump to developing a virtual reality clinical skill OSCE station is viable. Furthermore, using virtual reality would help to eliminate one of the biggest criticisms amongst medical students towards clinical skills stations: equipment bias across hospitals. By using a centrally standardised simulation, any mechanical or technical discrepancies between hospitals would be eliminated, making the stations fairer to students. Nevertheless, a big concern against a virtual reality clinical skills station is whether haptic technology combined with virtual reality is enough to suitably replace the process of actually performing the procedure, albeit on mannequins or plastic models. If the end goal is to be competent in completing procedures on real-life patients, can virtual reality ever be enough to simulate this? In response, one could argue that our present learning model of mannequins and plastic models is even less realistic and that the virtual experience would be more closely aligned to real-time patients.

For a communication-based OSCE station, it may be believed that nothing could be better than sitting face-to-face with another human being. Interestingly, however, virtual reality has become so advanced that the technology now exists which allows users to interact with computer-based virtual humans, simulating real-time conversations based on responses from both the user and the virtual patient ( Kron *et al*., 2017). The virtual humans encompass a full range of behaviours expected of two people talking together. Additionally, multiple studies now exist, assessing the ability of virtual reality to provide a suitable means of teaching communication skills ( Kleinsmith *et al*., 2015, Kron *et al*., 2017, Pan *et al*., 2016). Pan *et al*. used virtual reality to assess clinicians’ responses to a scenario involving a virtual patient demanding antibiotics, ensuring “difficult” communication ( Pan *et al*., 2016). The ability of virtual reality to simulate such scenarios suggests that its use for a communication-based OSCE station is possible. Furthermore, there are several advantages of assessing communication through virtual reality. Firstly, the need for actors would be eliminated. This, in turn, addresses one key issue often brought up by students. That is, the performance of actors might not be consistent from candidate to candidate throughout the day, inevitably varying due to human factors. In contrast, by using a virtual scenario, the behaviour of the computerised patient is both constant and reproducible, improving the face validity of the station. Secondly, virtual reality can be used to replicate scenarios where using a real patient is difficult or unethical such as paediatric cases or communication surrounding safeguarding ( Fertleman *et al*., 2018). Thus, expanding this, virtual reality can provide an opportunity to examine a variety of different communication scenarios that wouldn’t be present or possible within a normal OSCE station.

On the other hand, the study by Pan *et al* also demonstrates the potential barriers to implementing a virtual reality communication OSCE station. In the study, there was an operator selecting statements for the virtual patient’s response. There was no voice recognition software available to let communication be completely automatic ( Fertleman *et al*., 2018). For communication scenarios examining the user’s ability to ask questions, the ability to generate free-flowing communication could be negatively affected if responses have to be continuously programmed by an external operator. Another important consideration is the difficulty in trying to build interaction between the user and the virtual patient when the virtual patient lacks any emotive programming. With the present use of actors, although stations can still be scripted, communication is developed from both real-time response and emotion from both the candidate and the actor. Currently, technology still limits the virtual patient in this regard ( Marcos-Pablos *et al*., 2016).

## Conclusion

Virtual reality technology within the medical field has already shown incredible results, namely in the surgical setting. Now, within medical education, the outlook is truly promising. Of course, there is still a long way to go before virtual reality becomes a mainstay in any examination process. However, the potential for virtual reality making an impact within this field marks it an exciting vision for the future.

## Take Home Messages

• Virtual reality has been successfully implemented within the surgical field over the last decade, however, expansions into other areas of the medical profession are now being explored.
• Medical education and medical student training have the scope for integrating virtual reality technology and several studies have explored this.
• Advancements in virtual reality technology make its use as an assessment tool in OSCEs possible and there are advantages over conventional methods.
• Technological barriers and logistics still need to be overcome before virtual reality becomes a mainstay in assessments. Despite this, the outlook is both exciting and promising.

## Notes On Contributors

Arjuna Thakker is a final year medical student at the University of Birmingham. ORCID ID: https://orcid.org/0000-0001-5256-7939

Pooja Devani is an academic foundation year 1 doctor at Leicester Royal Infirmary, University of Leicester Hospital NHS Trusts. ORCID ID: https://orcid.org/0000-0002-5664-7888
