# Supplementary material for: Is there a role for virtual reality in objective structured clinical examinations (OSCEs)?
Source: MedEdPublish (2016). 2019 Sep 17;8:180. [Version 1] doi: 10.15694/mep.2019.000180.1 (PMC10712483; doi:10.15694/mep.2019.000180.1)
Supplement: Peer review history

# Reviewer report: Reviewer response for version 1

**DOI:** 10.21956/mep.19922.r30200

**Published:** 2019-12-04

**Authors:** Trudie Roberts

**Affiliations:** 1 Leeds Institute of Medical Education

This review has been migrated. The reviewer awarded 3 stars out of 5 I agree that VR/AR is an exciting development and could be used for OSCEs in the future but probably best used for scenarios which are otherwise difficult to simulate and which are crucial important such as dealing with emergency situations

Reviewer Expertise:

NA

No decision status is available

# Reviewer report: Reviewer response for version 1

**DOI:** 10.21956/mep.19922.r30201

**Published:** 2019-09-25

**Authors:** Trevor Gibbs

**Affiliations:** 1 AMEE

This review has been migrated. The reviewer awarded 4 stars out of 5 A very interesting discussion paper on an exciting but viable modern approach to the OSCE. The processes of development and enactment seem logical in this technology -advanced world of ours, but the authors clearly point out the advantages and pitfalls ahead. Having experienced some of the new virtual reality training models for dentistry at a recent simulation conference, I can see this approach to assessment developing. My only worry, and it is very personal, is the potential expense, which would lead to difficulties in resource -constrained countries.A definite read for all those involved in simulation training and assessment. I look forward to this developing.

Reviewer Expertise:

NA

No decision status is available

# Reviewer report: Reviewer response for version 1

**DOI:** 10.21956/mep.19922.r30199

**Published:** 2019-09-17

**Authors:** Felix Silwimba

**Affiliations:** 1 University of Lusaka

This review has been migrated. The reviewer awarded 5 stars out of 5 the article is informative and promising for the millennia learners. we are getting better.

Reviewer Expertise:

NA

No decision status is available
